# TomaFDNet: A multiscale focused diffusion-based model for tomato disease detection

**DOI:** 10.3389/fpls.2025.1530070

**Published:** 2025-04-24

**Authors:** Rijun Wang, Yesheng Chen, Fulong Liang, Xiangwei Mou, Guanghao Zhang, Hao Jin

**Affiliations:** ^1^ School of Teachers College for Vocational Education, Guangxi Normal University, Guilin, China; ^2^ Guangxi University Engineering Research Center of Agricultural and Forestry Intelligent Equipment Technology, Guangxi Normal University, Guilin, China

**Keywords:** deep learning, MSFDNet, objection detection, tomato disease, EPMSC

## Abstract

**Introduction:**

Tomatoes are one of the most economically significant crops worldwide, with their yield and quality heavily impacted by foliar diseases. Effective detection of these diseases is essential for enhancing agricultural productivity and mitigating economic losses. Current tomato leaf disease detection methods, however, encounter challenges in extracting multi-scale features, identifying small targets, and mitigating complex background interference.

**Methods:**

The multi-scale tomato leaf disease detection model Tomato Focus-Diffusion Network (TomaFDNet) was proposed to solve the above problems. The model utilizes a multi-scale focus-diffusion network (MSFDNet) alongside an efficient parallel multi-scale convolutional module (EPMSC) to significantly enhance the extraction of multi-scale features. This combination particularly strengthens the model's capability to detect small targets amidst complex backgrounds.

**Results and Discussion:**

Experimental results show that TomaFDNet reaches a mean average precision (mAP) of 83.1% in detecting Early_blight, Late_blight, and Leaf_Mold on tomato leaves, outperforming classical object detection algorithms, including Faster R-CNN (mAP = 68.2%) and You Only Look Once (YOLO) series (v5: mAP = 75.5%, v7: mAP = 78.3%, v8: mAP = 78.9%, v9: mAP = 79%, v10: mAP = 77.5%, v11: mAP = 79.2%). Compared to the baseline YOLOv8 model, TomaFDNet achieves a 4.2% improvement in mAP, which is statistically significant (P < 0.01). These findings indicate that TomaFDNet offers a valid solution to the precise detection of tomato leaf diseases.

## 1 Introduction

To meet the growing challenges faced by agricultural production due to global population growth and the intensification of climate change, smart agricultural technologies are increasingly recognized as essential tools to boost crop yields and ensure food security (Sanida et al., 2023). Among the world’s most widely cultivated cash crops, tomatoes play an important part in the food industry and daily diets. However, tomatoes are highly vulnerable to multiple diseases, which not only seriously reduce crop yields but also pose a threat to the sustainable development of global agriculture (Kaur et al., 2022). Early blight, late blight, and leaf mold are among the most frequently occurring and damaging diseases in tomato cultivation (Shanthi et al., 2024). If these diseases are not detected and treated promptly, they can lead to severe yield losses or even complete crop failure. Developing efficient and accurate methods for tomato disease detection is, therefore, critical for improving agricultural productivity and reducing economic losses.

Research on tomato leaf disease detection primarily falls into two categories: traditional manual feature extraction (Acharya et al., 2023; Narla and Suresh, 2023) and advanced deep learning approaches (Liang and Jiang, 2023; Lv and Su, 2024). Traditional methods rely heavily on manual identification by experienced farmers or agricultural experts, making the process time-intensive, labor-intensive, and often limited in accuracy due to subjective judgments and personal experience. Furthermore, the early symptoms of tomato diseases tend to be subtle, increasing the likelihood of missed detections or misdiagnoses in manual evaluations. Manual feature extraction methods typically involve image processing techniques to segment diseased areas, followed by the extraction of features such as color, shape, and texture to construct feature vectors for classification. For instance, Sabrol and Satish (2016) implemented an automatic classification approach utilizing color, shape, and texture attributes of tomato leaves. They applied Otsu’s segmentation algorithm in image preprocessing, followed by a classification tree algorithm, achieving an accuracy of 97.3% based on 24 features. Similarly, Jaisakthi et al. (2019) utilized the GrabCut segmentation algorithm to isolate leaves from the background, then employed a Support Vector Machine (SVM) for diagnosing diseases including grapevine black spot and leaf blight, thereby enabling more efficient disease identification.

In contrast, Convolutional Neural Network (CNN)-based deep learning approaches have exhibited remarkable potential in agricultural disease detection in recent years. These methods eliminate the need for manual feature extraction, allowing models to autonomously learn intricate image features, which greatly improves the precision and reliability of diseases identification. Recent advances in deep learning have shown significant progress in handling complex visual scenarios. For instance, techniques like Attentive GAN have demonstrated remarkable capabilities in processing challenging image conditions such as highlight removal from grayscale images (Xu et al., 2022), showcasing the potential of deep learning in addressing complex visual tasks. The target detection models utilizing deep learning can be generally categorized into one-stage and two-stage models. Classical one-stage models include SSD (Liu et al., 2016), RetinaNet (Lin et al., 2018) and YOLO series (Bochkovskiy et al., 2020; Qiao et al., 2020; Redmon and Farhadi, 2018, 2016; Wang et al., 2024a, b, 2022). While two-stage models comprise R-CNN (Girshick et al., 2014), Fast R-CNN (Girshick, 2015), Faster R-CNN (Ren et al., 2016) and Mask R-CNN (He et al., 2018). One-stage models integrate all detection steps within a single pipeline, directly predicting detection boxes and category labels from images, making them faster and ideal for instant processing. Two-stage models, however, split the process into two distinct stages: firstly, locating candidate zones and then classifying and refining these regions. While two-stage models generally achieve higher accuracy, they are computationally intensive and slower in processing.

For two-stage modeling, Zu et al. (2021) tackled the detection and segmentation of ripe tomatoes in complex environments using Mask R-CNN. They designed a mobile robot for automated image acquisition, integrating it with Mask R-CNN for target region detection and segmentation. Results demonstrated a high F1 score of 92.0% for both bounding boxes and mask regions, successfully detecting and segmenting tomatoes in greenhouse settings. Xie et al. (2020) developed the Faster DR-IACNN model, combining the Inception-v1 module, Inception-ResNet-v2 module, and Squeeze-and-Excitation Networks, enhancing multi-scale feature identification for grape leaf ailments. In another study, Syed-Ab-Rahman et al. (2022) proposed a two-phase convolutional neural network framework for identifying and classifying citrus diseases, employing a region proposal network (RPN) to detect affected areas and a subsequent classifier for disease classification. Gong and Zhang (2023) focused on detecting apple leaf diseases by introducing an enhanced version of Faster R-CNN, which incorporates Res2Net and a Feature Pyramid Network (FPN) to enhance multi-scale feature extraction capabilities, thereby allowing for precise identification of apple leaf diseases even in complex field environments.

On the other hand, You Only Look Once (YOLO), a renowned one-stage object detection algorithm, has gained popularity in agricultural applications due to its outstanding results on vast datasets like COCO. The YOLO algorithm is particularly favored for leaf disease detection tasks because of its speed and efficiency. Wang et al. (2024c) based on optimizing the TDGA model proposed by YOLOv5, by introducing global attention mechanism (GAM), switchable atrous convolution (SAConv) and EIoU, effectively solved the problem of shadow occlusion and small target detection of tomato leaf disease, enhancing detection accuracy by 2.93% relative to the original model. Liu et al. (2023) further refined the YOLOv5 algorithm, developing a method for detecting tomato brown rot that combines a hybrid attention mechanism with a CIOU loss function. This approach achieved a detection accuracy of 94.6% even in complex backgrounds, significantly enhancing the model’s disease recognition capabilities. Abdullah et al. (2024) utilized YOLOv8 for tomato leaf disease detection, applying data augmentation techniques on the PlantVillage dataset, which resulted in a mean Average Precision (mAP) value of 92.5%. This performance surpassed that of other models, including YOLOv5 and Faster R-CNN. Liu et al. (2022) introduced the YOLOv4-TAM algorithm, which integrates a triple attention mechanism with YOLOv4. This model optimized anchor frames using K-means clustering and introduced a focal loss function, leading to a significant increase in the recognition rate of tomato pests, achieving an accuracy of 95.2%. Wang and Liu (2024) proposed the TomatoDet model, which combines Transformer architecture with YOLOv8. This innovative approach leverages Swin-DDETR’s self-attention capability combined with the Meta-ACON dynamic activation to address the challenges of detecting small-target diseases, achieving a mAP value of 92.3%.

In summary, the aforementioned researchers have made significant strides in enhancing the precision and efficiency of tomato leaf disease detection. These advancements not only optimize model performance but also facilitate the development of intelligent agricultural disease detection technologies. However, despite notable progress, challenges remain in identifying targets across different scales. For instance, Sun et al. (2024) optimized the YOLOv8 algorithm for lightweight applications, achieving improvements in detection speed and overall performance. Nonetheless, the model’s capacity to detect targets at various scales, particularly small lesions in complex backgrounds, is still constrained, especially when addressing tomato lesions of varying sizes. Similarly, Liu et al. (2023) encountered limitations in fully resolving the detection issues associated with different scale targets while enhancing YOLOv5. The challenges were particularly evident in scenarios involving overlapping leaves and intricate lighting conditions, which hindered detection effectiveness. To address these shortcomings, this paper concentrates on optimizing the model’s multi-scale feature recognition capabilities. Building on the YOLOv8 algorithm, we introduce the Multi-Scale Focus-Diffusion Network (MSFDNet) and the Efficient Parallel Multi-Scale Convolution (EPMSC) module. This results in the proposed Tomato Focus-Diffusion Network (TomaFDNet) model specifically designed for tomato disease detection. The primary objective is to enhance detection accuracy, particularly for small target spots in complex backgrounds. The primary achievements of this paper are outlined below:

1. A novel TomaFDNet tomato disease detection model is proposed, and the accuracy of the model in detecting tomato diseases is enhanced through the integration of MSFDNet network and EPMSC module.
2. The MSFDNet network is specifically designed to effectively integrate multi-scale features and improve contextual semantic information through focusing and feature diffusion mechanisms. This network achieves accurate target detection, particularly excelling in recognizing multi-scale features. The network structure comprises feature focusing, diffusion, and fusion steps, which are combined with the Multi Scale Enhancer (MSE) module and the Cross-stage Partial Network with 2 convolutions (C2f) module to optimize feature extraction and refinement, ensuring accurate detection of targets across various scales.
3. The EPMSC module is proposed based on the concept of grouped convolution. By merging the design of grouped convolution with multi-scale convolution kernels, the model captures visual features at different scales more efficiently while maintaining computational efficiency, significantly enhancing its feature extraction performance.

The structure of the rest of this paper is as follows: In Section 2, we provide an overview of the dataset used and the methodology adopted for this research. Section 3 details the experiments performed and presents the results derived from the proposed methodology. Section 4 summarizes the study and explores potential directions for future investigation.

## 2 Materials and methods

### 2.1 Tomato disease dataset

**Figure 1:**
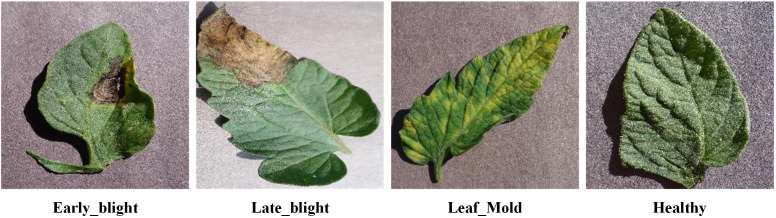
Examples of tomato disease dataset.

This study used the dataset called “Tomato Leaf Disease Detection with Global Attention” (TDGA), which comes from Wang et al. (2024c). It includes tomato diseases and is publicly accessible at https://github.com/zafucslab/TDGA. This dataset encompasses three prevalent tomato leaf diseases: Early_Blight, Late_Blight, and Leaf_Mold, in addition to images of healthy tomato leaves. Typical examples of these various types of diseased leaves are depicted in **Figure 1**. To meet the requirements of this study, the dataset was organized and reclassified accordingly. After thorough processing, the final dataset consisted of a total of 3,045 images. The images were allocated into training, validation, and test sets in an 8:1:1 proportion, with the exact count of images in each subset outlined in **Table 1**. This structured approach ensures that the model is trained and evaluated on a diverse and representative dataset, facilitating effective learning and robust performance in tomato disease detection.

**Table 1:** Image distribution of the dataset.

Disease type	Train	Validation	Test
Early_blight	770	99	101
Late_blight	404	44	53
Leaf_Mold	485	68	69
Healthy	777	93	82
Total	2436	304	305

**Figure 2:**
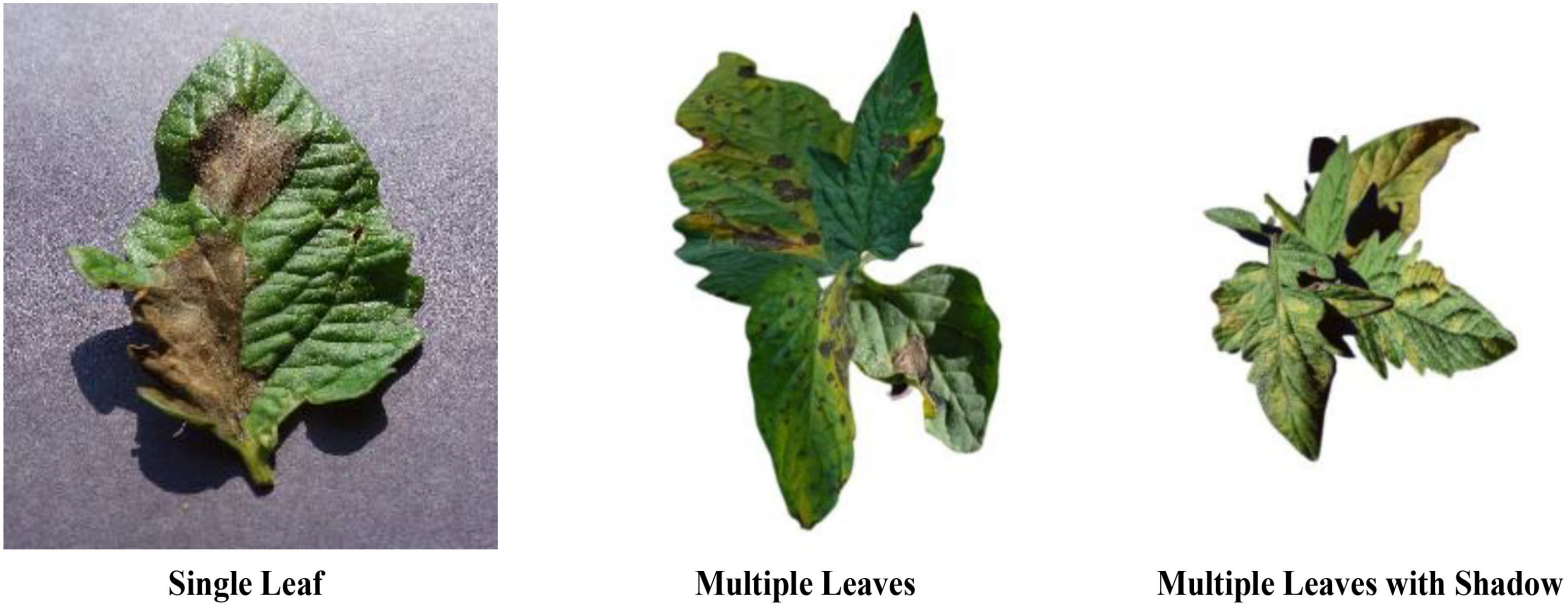
Examples of single-leaf, multiple-leaf, and shaded leaves.

In order to further assess the model’s ability to generalize and its stability, this study also acquired tomato leaf images from the Kaggle (Tomato Disease Multiple Sources) platform, encompassing diverse growing environments and shooting conditions. These images feature a variety of complex backgrounds and lighting conditions; however, they are not included in our primary dataset and are solely utilized for detection tests in varied scenarios to simulate real-world complexities. Additionally, we constructed datasets for multiple detection scenarios using the initial data. This includes images depicting single leaves, multiple leaves, and multi-leaf tomato leaf diseases, some of which feature shadows. The purpose of the method is to test the validity of the model in practical applications. Typical samples of these tomato leaf image, showcasing varying numbers of leaves and shadow effects, are illustrated in **Figure 2**. By incorporating these diverse scenarios, we aim to ensure the model’s steady and trustworthy operation in multiple real-life settings.

### 2.2 TomaFDNet network structure

**Figure 3:**
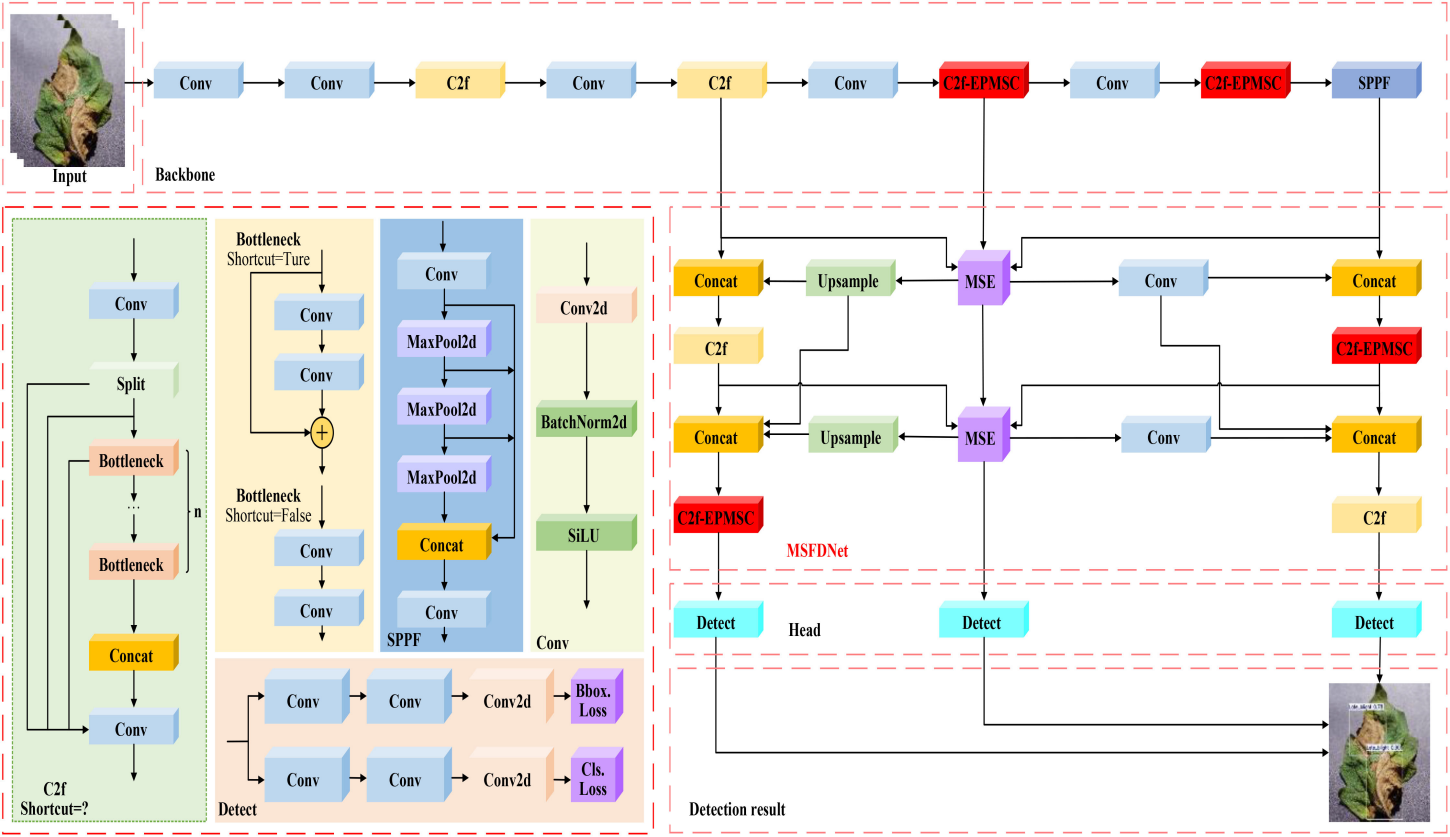
TomaFDNet network structure.

YOLOv8 is a target detection algorithm that leverages the advantages of its predecessors, YOLOv5 and YOLOv7. The framework comprises three primary elements: the backbone network, the neck architecture, and the detection head. The backbone network employs a lightweight design that integrates optimized ResBlocks with efficient convolutional operations, thereby reducing computational demands while enhancing feature extraction capabilities. YOLOv8’s neck structure enhances the model’s capacity to detect different-sized targets through efficient integration of multi-scale feature maps, while maintaining the spatial information’s completeness. Meanwhile, the detection head utilizes an anchor-free design that directly regresses the centroid and dimensions of the target. This simplification in model training reduces reliance on anchor parameters, contributing to YOLOv8’s widespread application. Despite these advantages, YOLOv8’s detection accuracy remains insufficient when faced with challenges posed by diverse scales, high similarity among targets, and complex backgrounds typical of tomato diseases. In order to overcome these limitations, we propose a novel tomato disease detection model, called TomaFDNet, which builds upon the YOLOv8 framework. The structure of TomaFDNet is illustrated in **Figure 3**. This model aims to enhance detection capabilities, particularly in recognizing tomato diseases across varying scales and under complex conditions.

TomaFDNet replaces the neck structure of YOLOv8 with the Multi-Scale Focused Diffusion Network (MSFDNet). This innovative approach, through a customized focusing module and diffusion mechanism, not only facilitates the extraction of richer feature information across multiple scales but also improves the network’s capacity to detect objects of different sizes, ultimately improving overall detection accuracy. Additionally, the C2f-EPMSC module is introduced to replace the original C2f module in YOLOv8. This modification optimizes the network’s feature extraction capabilities, enabling more efficient capture of multi-scale features. By employing grouped convolution strategies, we decrease the parameter count and computational burden, thereby enhancing both the computational efficiency and overall model efficacy. This design choice allows TomaFDNet to excel in the complex task of detecting tomato diseases across diverse conditions and target sizes.

### 2.3 Multi-scale focus-diffusion network

**Figure 4:**
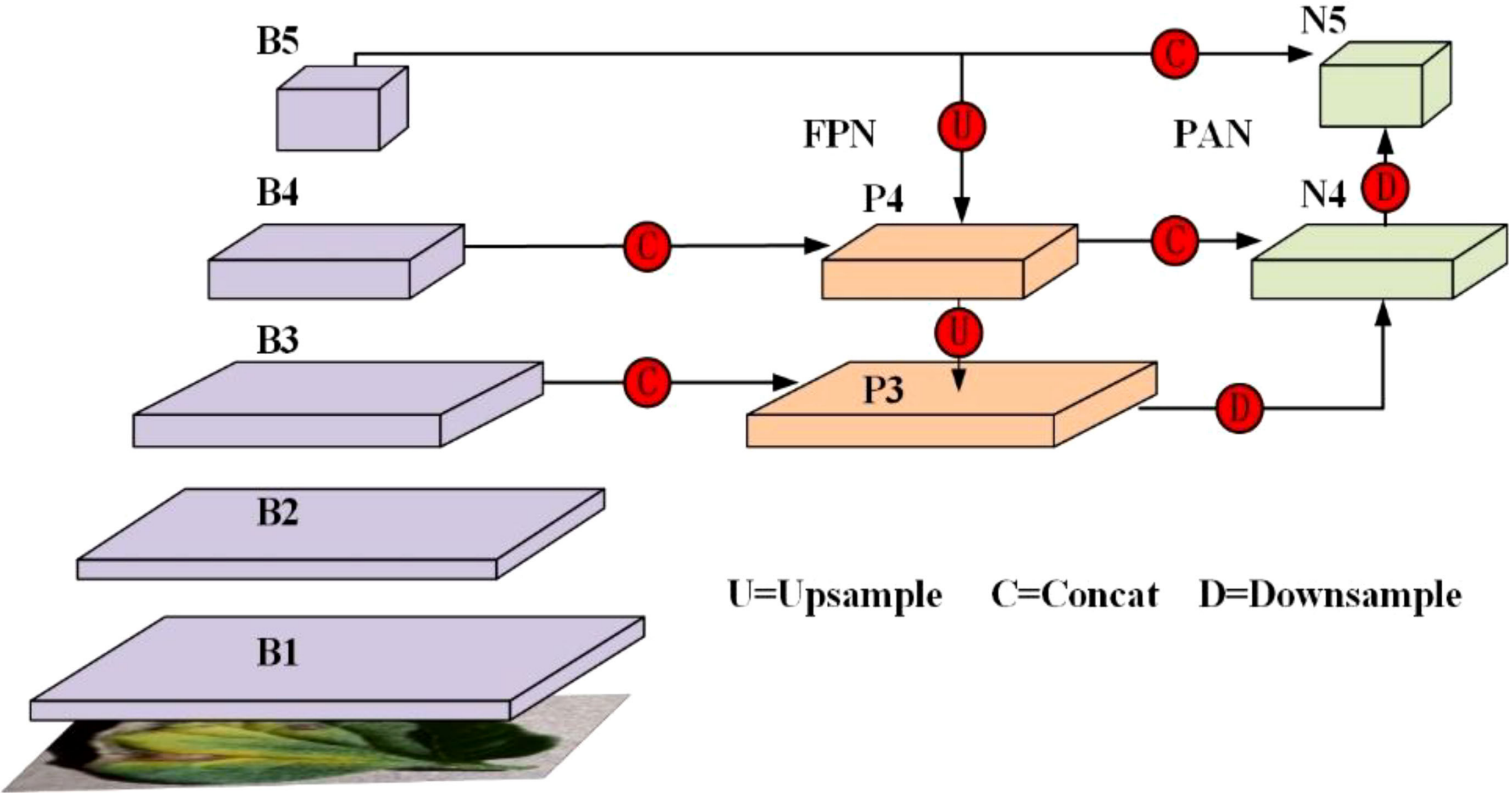
PAN-FPN structure in the YOLOv8 network.

In YOLOv8, the neck structure is built upon the integration of the Feature Pyramid Network (FPN) and the Path Aggregation Network (PAN), as illustrated in **Figure 4**. The primary function of the FPN is to facilitate the propagation of information among feature maps across different scales via top-down paths, enabling the integration of features from multiple scales. Specifically, the FPN transmits high-level semantic information down to lower-level feature maps via a layer-by-layer up-sampling process, subsequently merging these with corresponding low-level features. This approach effectively boosts the model’s target detection capability across different scales, particularly excelling in the identification of small targets.

However, this top-down feature propagation can sometimes result in the gradual dilution of high-level semantic features during the fusion process, potentially impacting detection accuracy in certain scenarios. To address this issue, YOLOv8 incorporates the PAN structure in addition to the FPN. The PAN progressively transmits local details from the low-level feature maps upwards through a bottom-up path propagation strategy, ultimately fusing these with the high-level feature maps. This bottom-up feature propagation compensates for the limitations of the FPN in small target detection, enabling the model to gather intricate details from low-level features more effectively, thereby improving the detection accuracy for small targets. This strategic enhancement in feature fusion is a critical aspect of the TomaFDNet model, which builds upon these principles to further improve multi-scale feature recognition and target detection accuracy in tomato leaf disease identification.

Although the integration of FPN and PAN demonstrates strong performance in various tasks, challenges remain, particularly in the context of small-target and contextually complex tasks like tomato leaf disease detection.

First, while the FPN effectively fuses high-level contextual information with low-level fine details during the up-sampling process, this fusion can lead to the cascading dilution of high-level semantic features. During up-sampling and lateral connections, the resolution of the feature map may increase; however, the intensity of high-level semantic features can be overshadowed by noise present in the low-level feature maps. This phenomenon hinders the model’s capacity to precisely identify small objects in cluttered scenes.

Second, although the PAN introduces a bottom-up path propagation strategy to enhance detail capture, it remains deficient in capturing global contextual information within the feature maps. The design of both FPN and PAN primarily focuses on the fusion of features across different scales, yet it does not fully leverage multisensory field information. This limitation becomes particularly evident when processing images with intricate textures and backgrounds.

Finally, in the context of tomato leaf disease detection, lesions are often characterized by subtle color and texture variations. The existing FPN and PAN structures struggle to integrate these fine-grained features effectively. While the PAN can convey fine details extracted from lower-level features, these details are frequently lost when combined with higher-level features because of the lack of multi-receptive field processing capabilities. This deficiency ultimately impacts the accurate identification of disease spots, highlighting the need for a more robust approach to feature integration and recognition in complex scenarios.

Based on the analysis of the limitations inherent in the YOLOv8 neck structure for tomato leaf disease detection, we propose a novel neck structure named Multi-Scale Focus-Diffusion Network (MSFDNet) to supplant the original FPN-PAN configuration. The primary concept of MSFDNet revolves around the focused processing of feature maps at multiple scales, complemented by a diffusion mechanism that propagates the information derived from these focused feature maps across different scales.

**Figure 5:**
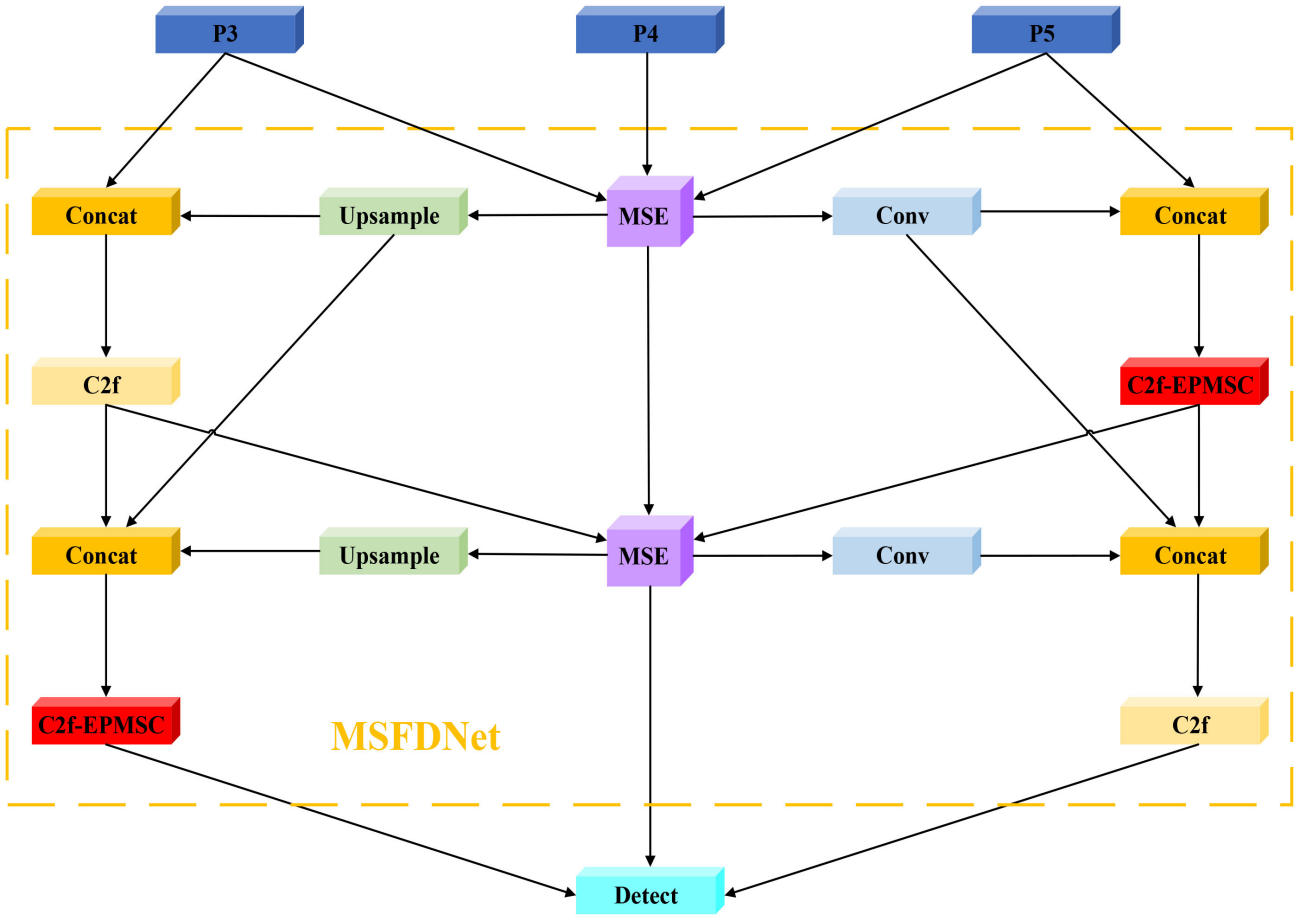
Structure of the MSFDNet.

The overall architecture of MSFDNet is composed of multiple stages, each encompassing three key steps: feature focusing, feature diffusion, and feature fusion. This design allows the network to seamlessly combine data from various scales, thereby enhancing the contextual semantic information of the features. As a result, MSFDNet is capable of achieving more precise target detection in complex scenes, particularly when addressing targets characterized by multi-scale features. The structure of MSFDNet is illustrated in **Figure 5**, showcasing its innovative approach to feature processing and integration, which aims to improve the model’s effectiveness at tomato leaf disease detection by addressing the challenges associated with diverse scales and complex backgrounds.

The MSFDNet framework incorporates a structure akin to a feature pyramid, augmented with the Multi-Scale Enhancer (MSE) module, whose details are discussed further in Section 2.4. The core workflow of MSFDNet can be outlined in several key stages:

1. **Focused Multi-Scale Feature Extraction**: The network initially processes the multi-scale feature maps (P3, P4, P5) produced by the main network through the MSE module. This module produces a more concentrated multi-scale feature representation, serving as the foundation for feature focusing and diffusion. The MSE module effectively captures essential information from input features across varying scales while preserving rich contextual semantics.
2. **Feature Fusion Across Scales**: MSFDNet then combines multi-scale feature maps via numerous upsampling and downsampling operations. These feature maps are concatenated via Concat operations, thereby integrating information across various spatial resolutions and yielding richer feature representations.
3. **Enhanced Feature Refinement with C2f and C2f-EPMSC Modules**: After feature fusion, MSFDNet utilizes YOLOv8’s original C2f module alongside the newly designed C2f-EPMSC module for further feature enhancement. This refinement involves blending features across spatial and channel dimensions, which increases feature diversity and improves target recognition.
4. **Iterative Focusing and Diffusion**: The network iteratively updates and enhances the feature map through repeated focusing and diffusion processes. This ensures that at the end of each stage, feature maps across different scales are enriched with more robust contextual information.
5. **Detection via Multi-Scale Input**: After multiple focusing and diffusion iterations, the final multi-scale feature maps are processed by the Detect module, which takes inputs from the P3, P4, and P5 scales. This multi-scale input enables the model to effectively detect target objects across various scales, ensuring that detection performance is robust even in scenarios with complex or overlapping objects.

Overall, MSFDNet strengthens multi-scale feature integration and contextual understanding, addressing challenges in identifying disease spots of varying scales in intricate backgrounds.

### 2.4 Multi scale enhancer

The Multi-Scale Enhancer (MSE) module is a key feature fusion component within the MSFDNet network, designed to enhance multi-scale feature fusion and processing. This module consists of five main parts: upsampling, a downsampling layer, a depthwise convolutional layer (DWConv) with multiple kernels, a pointwise convolution (PWConv) layer, and a residual connection. The inputs to the MSE module are three feature maps of different resolutions: *X*_1_, *X*_2_ and *X*_3_. Features *X*_1_ and *X*_2_ are adjusted via upsampling and downsampling, respectively, using conventional convolution to maintain consistent channel numbers. For downsampling *X*_3_, we introduced the Adown (Wang et al., 2024b) module, which leverages max pooling and average pooling operations to capture diverse contextual information, thereby generating enhanced features through downsampling.

**Figure 6:**
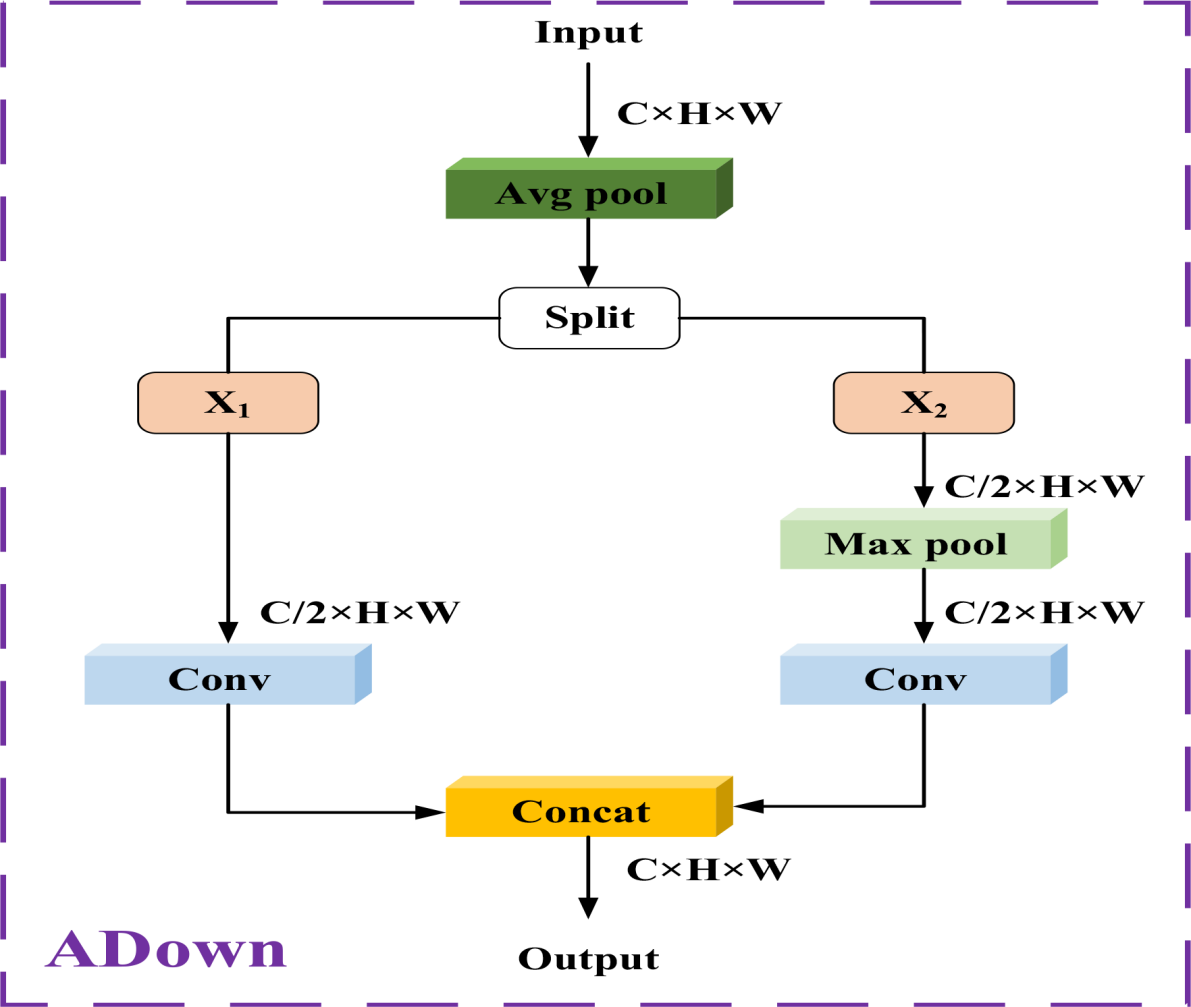
Structure of the ADown model.

As illustrated in **Figure 6**, the ADown module begins by applying average pooling to the feature map *X.* This pooling operation reduces noise by averaging adjacent regions, capturing an overall representation of the input features. After average pooling, the feature map *X* is divided along the channel dimension into *X*_1_ and *X*_2_, allowing the module to apply different processing strategies to each part and thus retain more spatial information. The difference between *X*_1_ and *X*_2_ lies in an additional max pooling applied to *X*_2_, which helps retain critical information within the feature map. Following these operations, both feature maps undergo convolution processing and subsequently fused together along the channel dimension, resulting in a new feature map. Through these steps, the ADown module not only reduces spatial dimensionality but also minimizes information loss during downsampling, providing a richer feature representation for subsequent processing.

**Figure 7:**
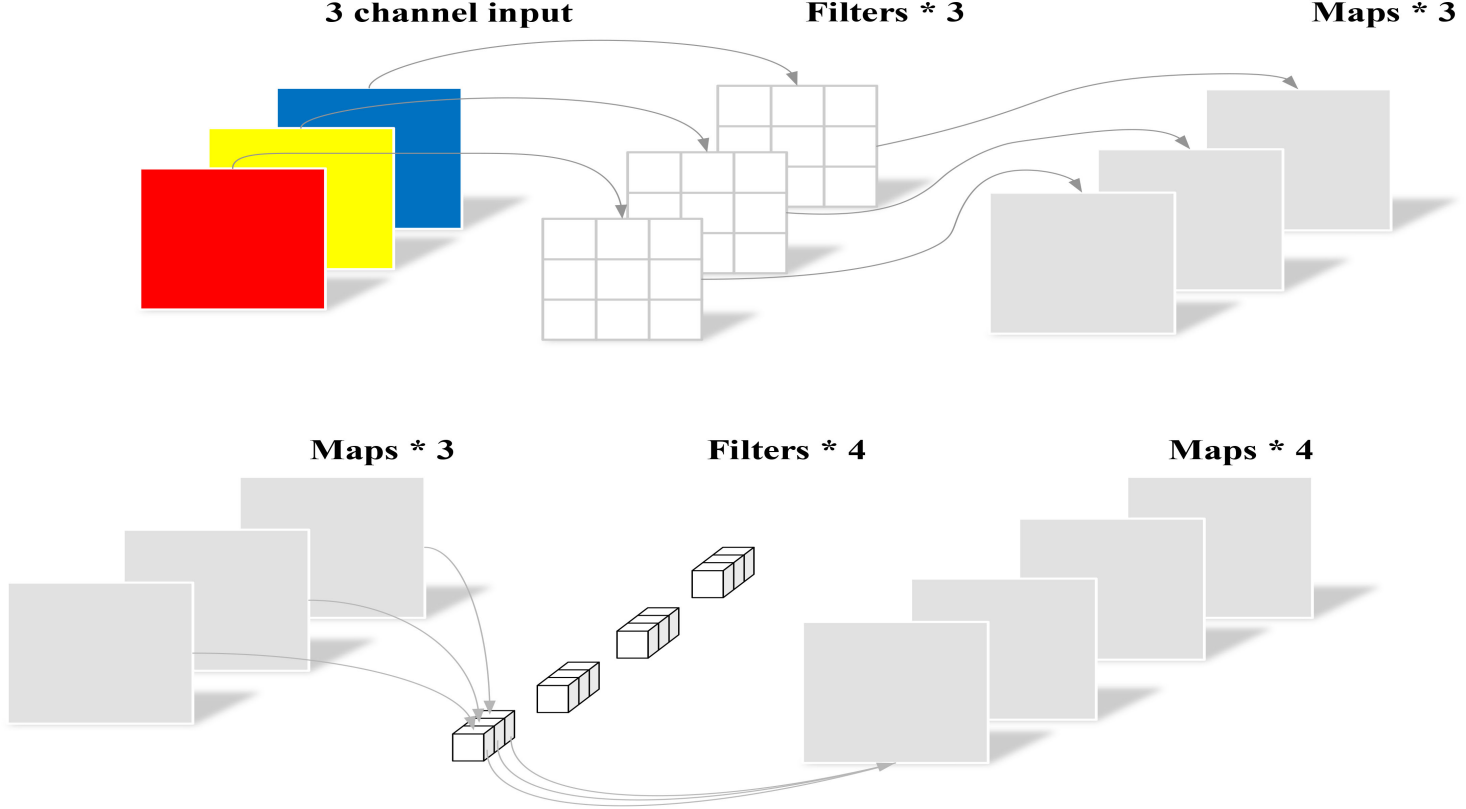
Structure of depthwise separable convolutions. top: DWConv; bottom: PWConv.

Next, Depthwise Separable Convolutions (Chollet, 2017) are applied to the scaled-adjusted feature maps, which are processed by deep convolutions of varying kernel sizes (5×5, 7×7, 9×9, 11×11) in parallel. Each convolution kernel is designed to capture essential information within distinct receptive fields, providing a more nuanced feature representation. Following these operations, the outputs from each kernel are fused using a pointwise convolution layer, resulting in a more comprehensive feature map. The illustration of Depthwise Separable Convolutions’ structure is provided in **Figure 7**.

**Figure 8:**
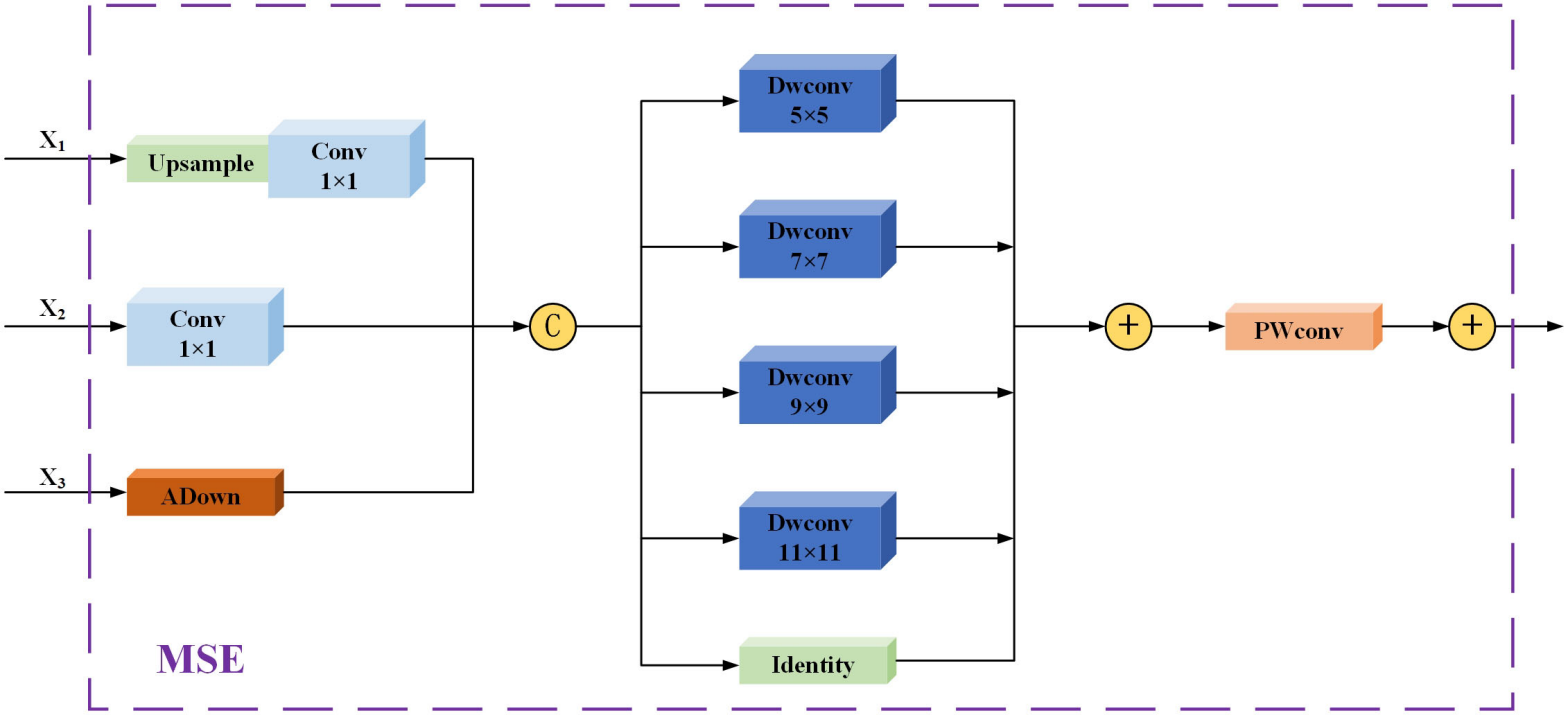
Network Structure of MSE.


Finally, to preserve the integrity of the original feature map and enhance the representation of new features, the module combines the original and enhanced features via a residual connection, outputting the final multi-scale enhanced feature map. By integrating the Depthwise Separable Convolutions mechanism, the MSE module significantly improves MSFDNet’s performance in complex scenes and for subtle object detection. Its efficient strategy for feature enhancement and fusion allows MSFDNet to effectively integrate information across feature maps of varying scales, thereby greatly enhancing the precision and reliability in detecting targets. This design effectively addresses the limitations of traditional FPN and PAN structures in complex scenarios. **Figure 8** illustrates the structure of the MSE module. MSE is detailed in Equation 1:

(1) $$\begin{array}{c} Y=X_{conv1}+PWConv(\sum_{k\in kernels}DWC_{k}(Concat(X_{conv1},X_{conv2},X_{ADown}))) \end{array}$$

In the formula, $X_{conv1}$ is the feature graph of input feature $X_{1}$ after up-sampling and convolution processing, $X_{conv2}$ is the feature graph of input feature $X_{2}$ after convolution processing, and $X_{ADown}$ is the feature graph of input feature *X*_3_ after ADown downsampling processing. Concat Indicates the concatenation operation of feature maps. $DWC_{k}$ represents deep convolution operations with different kernel sizes. PWConv is the pointwise convolution operation, and *Y* is the enhanced feature after fusion.

### 2.5 Efficient parallel multi-scale convolution

#### 2.5.1 GhostConv grouping idea

**Figure 9:**
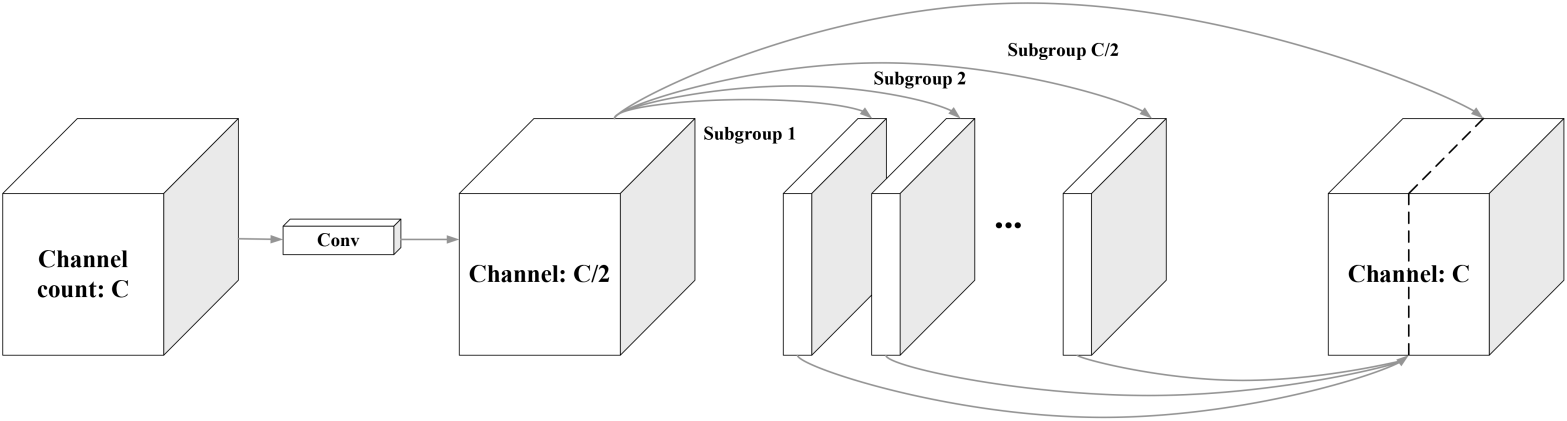
Ghost convolution.

Traditional convolutional feature extraction in object detection models often results in redundant information, increasing the computational complexity. To address this, the lightweight network GhostNet, proposed by Han et al. (2020), leverages grouped convolution to eliminate unnecessary feature information, reducing model parameters and enhancing detection speed. The core innovation of GhostNet, known as “GhostConv”, generates more feature maps with fewer computations, effectively capturing essential features while maintaining a compact model size. GhostConv accomplishes this by splitting the input channels into smaller groups and applying simple linear operations to produce “ghost” features, subsequently combined with the initial feature map. The GhostConv principle is illustrated in **Figure 9**.

In Ghost convolution, the input feature map first generates a small set of base feature maps (Subgroup 1) using a standard convolution operation. These foundational feature maps are then subjected to a series of simple linear transformations to create additional ghost features (Subgroup 2). The ghost features are subsequently merged into the base feature map to yield the final output feature map, as illustrated in **Figure 9**.

The parameter count for GhostConv is defined as follows: let the number of input channels be *C*, and the convolution kernel sizes for the grouped convolutions be *K*_1_ and *K*_2_, respectively, with the number of groups denoted by *g*. The total parameters *P_g_* of GhostConv can be expressed as by Equation 2:


(2) $$\begin{array}{c} P_{g}=\frac{K_{1}^{2}\times C}{2}+\frac{K_{2}^{2}\times C}{2}+\frac{C^{2}}{g} \end{array}$$

Where the first and second terms: $\frac{K_{1}^{2}\times C}{2}+\frac{K_{2}^{2}\times C}{2}$ represent the number of parameters in the grouping convolution, and the third term: $\frac{C^{2}}{g}$ represents the parameters that generate the ghost feature (subgroup2) by linear operation from the base feature graph (subgroup1). In contrast to the standard convolution, the number of output channels is $C^{\prime }$ and the convolution kernel size is *K*. The number of standard convolution parameters $P_{c}$ is given by Equation 3:


(3) $$\begin{array}{c} P_{c}=K^{2}\times C\times C^{\prime } \end{array}$$

In the formula, all channels are engaged in convolution operations, resulting in high computational effort. To compare the efficiency of Ghost convolution with that of standard convolution, the ratio of their parameter count reduction can be determined by Equation 4:


(4) $$\begin{array}{c} \frac{P_{g}}{P_{c}}=\frac{K_{1}^{2}+K_{2}^{2}}{2K^{2}\times g}+\frac{1}{g} \end{array}$$

This formula demonstrates that Ghost convolution significantly reduces the parameter count, particularly when the count of groups *g* is large, making the reduction effect even more pronounced.

#### 2.5.2 C2f-EPMSC module

Inspired by the concept of grouped convolution, we designed a module named C2F-Efficient Parallel Multi-Scale Convolution (C2f-EPMSC) to serve as a replacement for the original C2f module of the YOLOv8 algorithm. C2f-EPMSC is a novel lightweight multi-scale convolution module that integrates grouped convolution with multi-scale convolution kernels. This design enables more efficient capture of multi-scale features while maintaining computational efficiency and improving the model’s ability to extract features.

**Figure 10:**
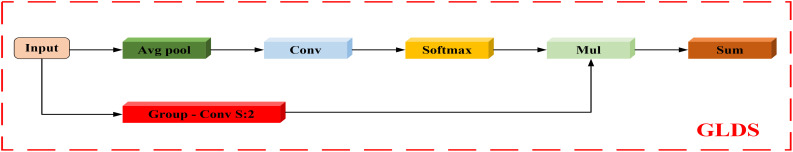
Network structure of GLDS.

The EPMSC module employs a Grouped Lightweight Downsampling (GLDS) structure, as illustrated in **Figure 10**. This module implements a lightweight adaptive weight downsampling procedure. Initially, the input feature map goes through an average pooling layer followed by a 1×1 convolution layer to generate a spatial attention map. This attention map then performs a rearrangement operation to map the 2×2 regions to a new dimension, normalizing it along the last dimension using the Softmax function to derive the weights for each subregion. Subsequently, the input feature map is downsampled using a grouped convolution layer, resulting in an output channel count that is four times the original. The subsampled feature map is rearranged to group every four channels into a new dimension. Ultimately, the rearranged feature map is assigned weights and multiplied by the previously generated weight map, and the weight dimension is summed to produce the final downsampling result.

**Figure 11:**
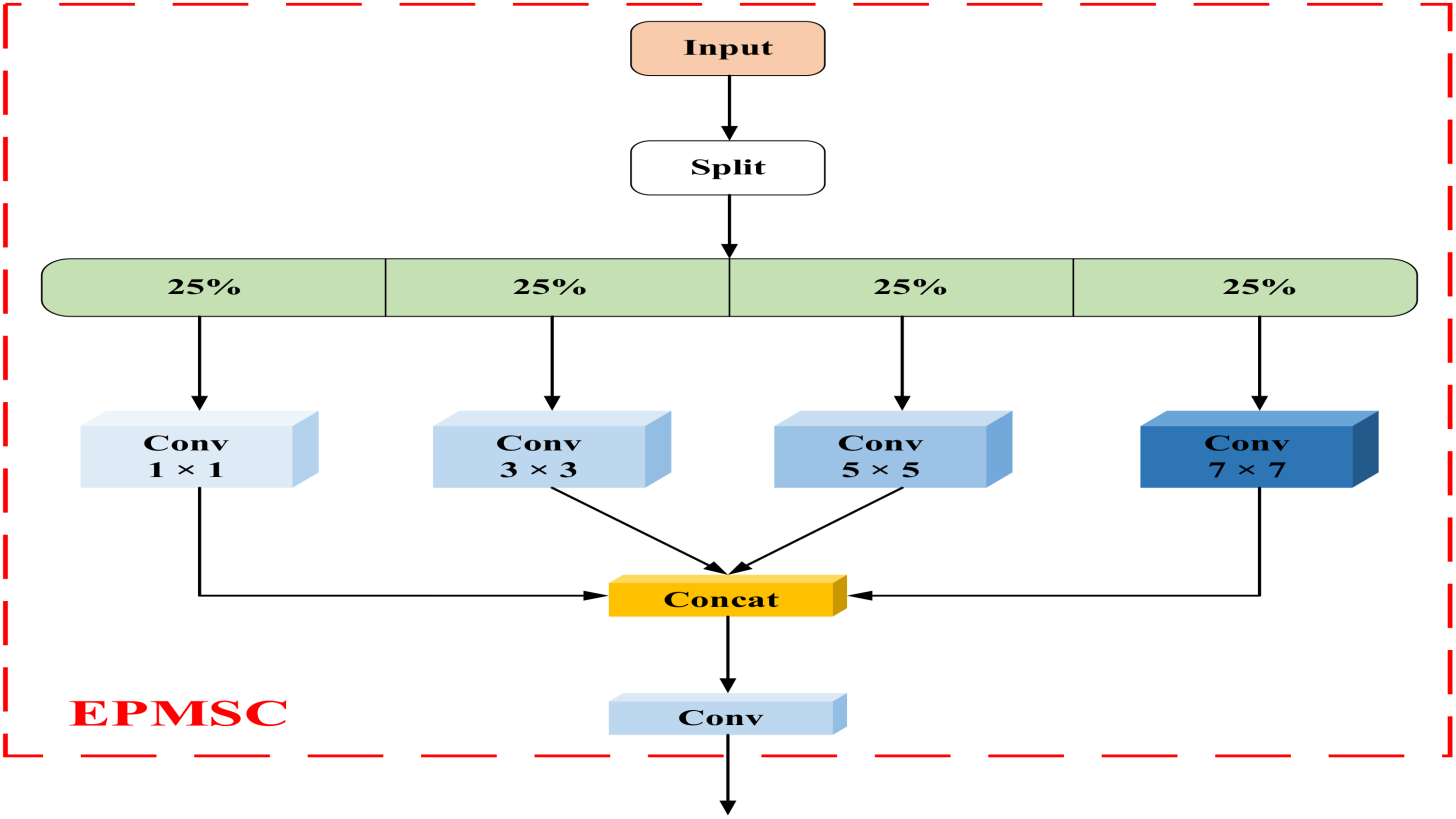
Network structure of EPMSC.

In the EPMSC module, input features are grouped, and each group is convolved independently using convolution kernels of varying sizes (1×1, 3×3, 5×5, and 7×7). This grouping strategy significantly reduces both the parameter count and computational demands, while preserving the diversity and expressiveness of the features. This structure enables different convolution kernels to operate on distinct sets of channels, allowing each kernel to concentrate on extracting features at a specific scale without interfering with the operations of others. **Figure 11** provides an illustration of the structure of the EPMSC module.

The EPMSC module works as follows:

1. **Feature Splitting**: Initially, the input feature map is segmented into four distinct channel groups through a “Split” operation, with each group representing 25% of the total input channels. This grouping prepares the data for subsequent multi-scale convolution, enabling each set to be processed independently.
2. **Multi-Scale Convolution**: Each group is then convolved with a different kernel size (1×1, 3×3, 5×5, and 7×7). Smaller kernels, such as 1×1, focus on fine-grained local details, whereas larger kernels, like 7×7, capture wider contextual information. This multi-scale approach allows the module to adapt effectively to different spatial patterns within the feature map, enhancing both the perceptual range and generalization ability of the network.
3. **Feature Concatenation**: After convolution, the processed feature maps from each kernel size are combined through a “Concat” operation along the channel dimension, restoring the feature map to its original channel count. This step ensures that the information gathered from each scale is preserved and accessible for subsequent processing.
4. **Final Integration with 1x1 Convolution**: The concatenated feature map is subsequently processed with a 1×1 convolution. This operation reduces redundant information while enhancing the interaction between features at different scales, creating a more cohesive multi-scale representation.
5. **Output Generation**: The final feature map, enriched with information from multiple receptive fields, is now ready for subsequent layers. By leveraging both grouping and multi-scale convolution, the EPMSC module effectively captures diverse spatial features and improves detection accuracy, particularly in tasks involving small objects and complex backgrounds.

### 2.6 Method evaluation

We selected average precision (AP) and its mean, mean average precision (mAP), as the evaluation metrics to assess the performance of the TomaFDNet model. These metrics are commonly utilized in object detection tasks. The formulas for each evaluation indicator are detailed in Equations 5–8


(5) $$\begin{array}{c} Precision=\frac{TP}{TP+FP} \end{array}$$

(6) $$\begin{array}{c} Recall=\frac{TP}{TP+FN} \end{array}$$

(7) $$\begin{array}{c} AP=\int_{0}^{1}P(R)dR \end{array}$$

(8) $$\begin{array}{c} \text{mAP}=\frac{\sum_{i=1}^{c}AP_{i}}{C} \end{array}$$

Where, TP (True Positive) represents the count of samples that are accurately identified as having tomato disease, whereas FP (False Positive) indicates the number of samples that are erroneously labeled as diseased when they are actually healthy. FN (False Negative) represents the samples misclassified as background when they exhibit tomato disease. Precision-recall (P-R) curve’s area is quantified by AP which reflects the relationship between precision (P) and recall (R). On the other hand, mAP is determined by averaging the AP values for all disease categories, not the optimal value for a single type of disease. mAP is an overall measure of model performance.

## 3 Results and discussion

In this section, we assessed the performance of the TomaFDNet model by analyzing its effectiveness in detecting tomato diseases. To evaluate the performance of the TomaFDNet model, we used a publicly available tomato leaf disease dataset. The dataset contains three common tomato Leaf diseases: Early Blight, late Blight, Leaf Mold, and images of healthy tomato leaves. The total number of images in the dataset is 3045, and the dataset is divided into training set, verification set and test set, with a ratio of 8:1:1. During model training and prediction, we maintained consistent hyperparameter settings across all experiments, which included a batch size of 16, 28 workers, and 200 epochs. To guarantee the reliability and comparability of the experimental outcomes, all experiments were conducted in a uniform hardware and software environment. Detailed configurations of the experimental platform and training parameters are presented in **Tables 2** and **3**.

**Table 2:** Experimental environment setup details.

Device name	Model or Name	Parameter or version
GPU	NVIDIA GeForce RTX 4060 Laptop	Video Memor:8GB
CPU	13th Gen Intel(R) Core(TM) i9-13900HX	frequency:2.20 GHz
Computer operating system	Windows 11	Internal memory:32GB
Development environment software	PyCharm	2023.2.1
Programming language	Python	3.8
Deep learning framework	PyTorch	2.1.0
Computational acceleration	CUDA	12.1

**Table 3:** Hyperparameter settings for experiments.

Hyperparameters	Configurations
Image Dimensions	640
Batch size	16
Workers	28
Epoch	200
Learning rate	0.01
Momentum	0.937
Weight_decay	0.0005
Close_mosaic	0
Device	0
Optimizer	SGD
Cache	False

### 3.1 Ablation experiment

This section primarily evaluates the effectiveness of the proposed MSFDNet structure and EPMSC module for tomato disease detection. We conducted ablation experiments based on the YOLOv8 algorithm, incorporating two improvements: the MSFDNet structure and the EPMSC module, into the original YOLOv8 framework. The experimental results are summarized in **Table 4**. Here, “YOLOv8” refers to the original YOLOv8 algorithm, “YOLO+MSFDNet” indicates that the neck structure of YOLOv8 has been replaced with the MSFDNet structure, and “YOLO+EPMSC” signifies the incorporation of the EPMSC module in place of the C2f module. Finally, “TomaFDNet” represents the combination of both the MSFDNet structure and the EPMSC module integrated into the YOLOv8 framework.

**Table 4:** Results of ablation experiment.

Methods	mAP	AP			
		Early_blight	healthy	Late_blight	Leaf_Mold
YOLOv8	0.789	0.686	0.994	0.826	0.649
YOLOv8 + MSFDNet	0.807	0.685	0.992	0.871	0.679
YOLOv8 + EPMSC	0.799	0.689	0.992	0.872	0.642
TomaFDNet	0.831	0.708	0.993	0.870	0.753

The findings displayed in **Table 4** demonstrate that the introduction of the MSFDNet and EPMSC modules significantly enhances the mAP compared to the original YOLOv8 model, with increases of 1.8% and 1%, respectively. When both modules are incorporated simultaneously, the mAP reaches 83.1%, representing an overall improvement of 4.2%. These findings indicate that the proposed model effectively enhances the accuracy of tomato leaf disease detection.

To demonstrate the superiority of the MSFDNet structure in tomato disease detection, we conducted comparative experiments with several mainstream neck structures, including the original YOLOv8’s FPN-PAN, BiFPN (Tan et al., 2020), MAFPN (Yang et al., 2024), HSFPN (Chen et al., 2024) and EfficientRepBiPAN (Li et al., 2022). The results are displayed in **Table 5**. In this table, “YOLOv8” refers to the original network without any modifications, which utilizes the FPN-PAN structure as its neck. “YOLOv8+BiFPN” indicates that the neck structure of YOLOv8 has been replaced by BiFPN, with the remaining structures named similarly.

**Table 5:** Comparison of replacement mainstream neck structures.

Methods	mAP	AP			
		Early_blight	healthy	Late_blight	Leaf_Mold
YOLOv8(FPN-PAN)	0.789	0.686	0.994	0.826	0.631
YOLOv8 + BiFPN	0.797	0.68	0.992	0.847	0.671
YOLOv8 + MAFPN	0.796	0.701	0.993	0.837	0.654
YOLOv8 + HSFPN	0.799	0.684	0.99	0.85	0.673
YOLOv8 + EfficientRepBiPAN	0.782	0.667	0.99	0.828	0.642
YOLOv8 + MSFDNet	0.807	0.685	0.992	0.871	0.679

Based on our examination of the experimental outcomes, we reach the following conclusions:

1. Replacing the neck structure of the YOLOv8 algorithm with BiFPN resulted in a 0.8% increase in mAP. The enhancement is due to BiFPN’s capacity for adaptive weighted fusion of feature layers, enabling two-way information flow by facilitating the passage of information from lower-level to higher-level features, while also allowing feedback from higher-level features to lower-level ones. However, the additional weighting and fusion operations required by BiFPN increase computational costs, consequently reducing the model’s inference speed.
2. The MAFPN structure improved mAP by 0.7%, enhancing the extraction capability for small targets and multi-scale features through shallow and deep information interactions in multi-branch assisted fusion (MAFPN). While this enhancement contributes to greater accuracy, it may also render the model overly sensitive to specific details, leading to potential overfitting on small targets in noisy data or under complex lighting and background conditions, thereby affecting the model’s generalization ability.
3. The HSFPN structure yielded a 1% increase in mAP. By introducing a hierarchical adaptive fusion strategy, HSFPN can flexibly adjust the feature fusion weights based on the resolution and semantic information of different feature layers. Although the adaptive weighting mechanism of HSFPN can effectively utilize feature maps of varying scales in ideal conditions, the model may struggle to adjust weights correctly in complex background scenarios due to noise interference.
4. The application of the EfficientRepBiPAN improved structure resulted in a 0.7% decrease in mAP. While the efficiency of feature fusion is enhanced, this lightweight design may compromise the model’s ability to extract small-scale targets and detailed features. Given that tomato disease detection requires heightened detail sensitivity, the EfficientRepBiPAN structure may inadequately capture critical information, leading to a decline in mAP values.
5. By replacing the YOLOv8 neck structure with our proposed MSFDNet structure, we achieved a notable increase in mAP of 1.8%, outperforming all other comparison structures. This enhancement is attributed to the aggregation and diffusion concept inherent in the MSFDNet structure, which effectively integrates information across different scales through multiple upsampling and downsampling operations. This approach not only preserves the semantic information of each scale but also enhances the ability to capture fine-grained features, significantly improving detection accuracy. Furthermore, MSFDNet reinforces the contextual information of feature maps through repeated focusing and diffusion processes, ensuring comprehensive information flow and fusion between multi-scale feature maps.

From the analysis above, it is evident that the original YOLOv8 algorithm, utilizing the FPN-PAN structure, achieves a mAP of 78.9%. The mAP decreased by 0.7% following the implementation of the EfficientRepBiPAN structure. Conversely, upon replacing the neck structure with BiFPN, MAFPN, and HSFPN, the mAP improved by 0.8%, 0.7%, and 1%, respectively. Although these three structures demonstrated slight enhancements in detection performance, the improvements were minimal compared to the substantial gains achieved with the MSFDNet structure. The experimental results and subsequent analysis clearly indicate that the MSFDNet structure outperforms other mainstream neck structures, effectively enhancing the accuracy of tomato disease detection. Consequently, we have integrated the MSFDNet structure into our proposed model.

### 3.2 Performance comparison experiments of different detection networks

In this performance comparison, we conducted comparative experiments using the same training set, test set, and environmental configuration parameters across various target detection models. This approach allowed us to further validate the superior performance of the TomaFDNet model in detecting tomato diseases.

**Figure 12:**
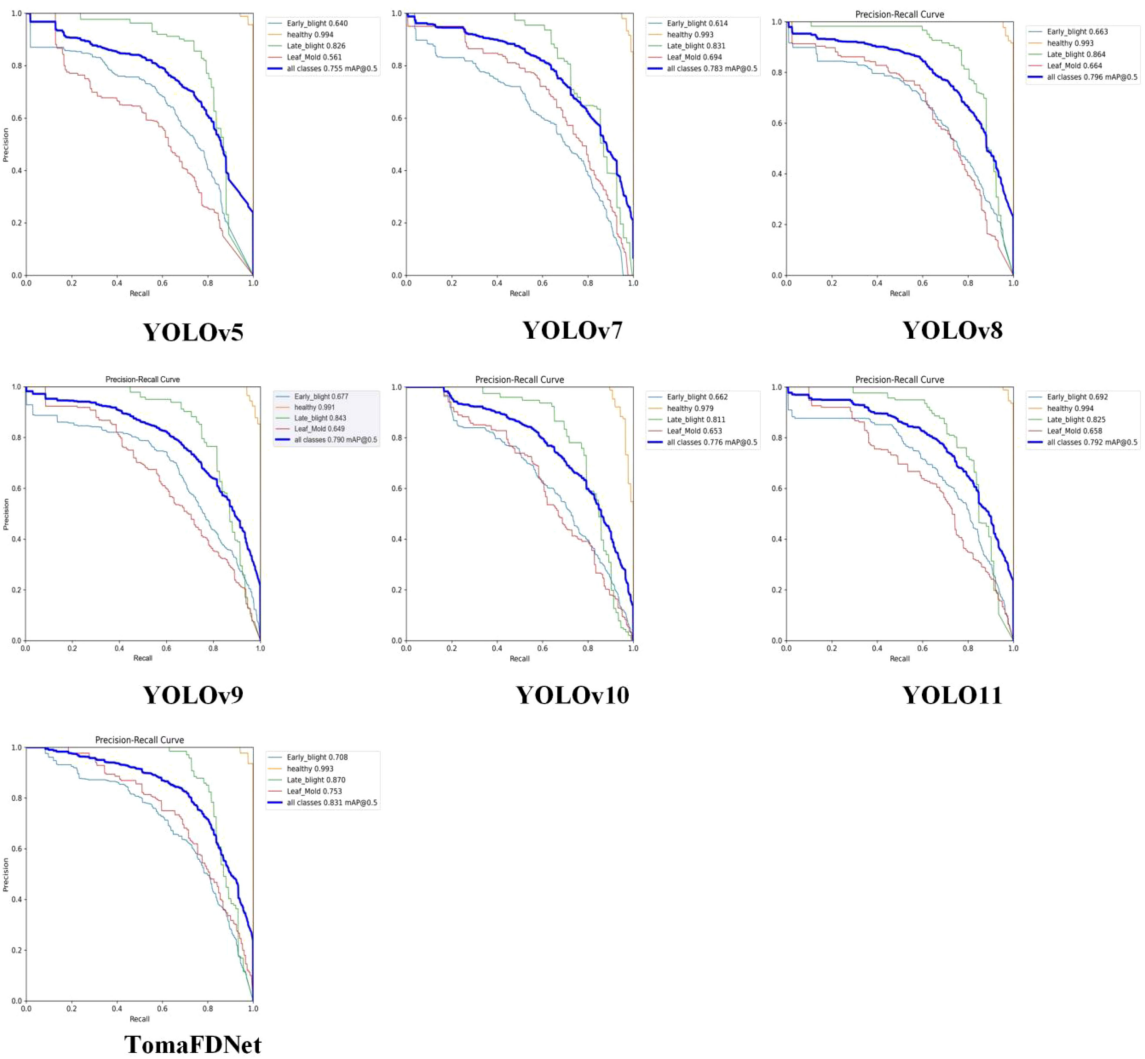
P-R curve of YOLO series algorithm.

**Figure 12** illustrates the P-R curves for various YOLO series target detection algorithms applied to the tomato disease test set. The PR curve is utilized to assess the models’ comprehensive performance at various thresholds in the object detection task, highlighting the relationship between precision and recall, thereby reflecting the models’ ability to manage positive and negative samples. AP can be understood as the area under the PR curve and is an indicator of performance detection. The higher the AP value, the better the performance. As depicted in **Figure 12**, the P-R curves for YOLOv5, YOLOv7, YOLOv8, YOLOv9, YOLOv10, YOLO11, and TomaFDNet are presented. Notably, the TomaFDNet model outperforms the other models in detecting the three tomato diseases. Specifically, the TomaFDNet model exhibited increased AP values by 2.2% for Early Blight, 4.4% for Late Blight, and 10.4% for Leaf Mold, in comparison to the original YOLOv8.

**Figure 13:**
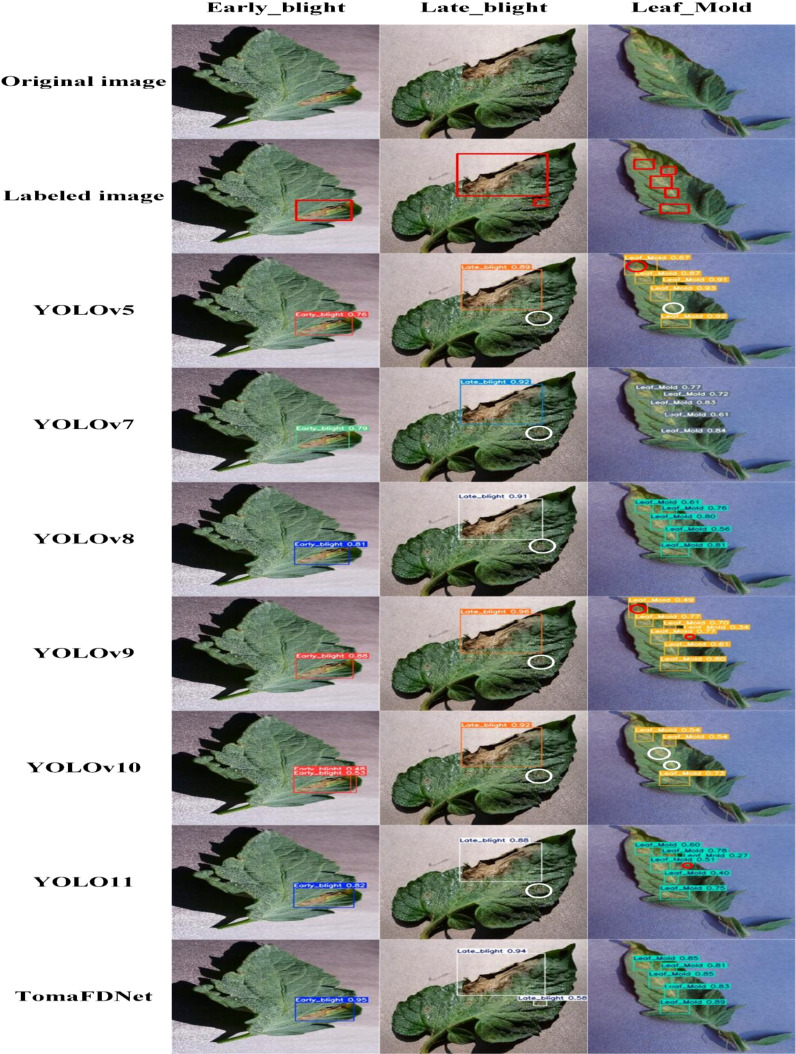
Comparison of detection results.

To provide a more intuitive demonstration of the TomaFDNet model’s performance in tomato disease detection tasks, we conducted a visual comparison experiment, with results displayed in **Figure 13**. This figure presents the actual prediction outcomes of the YOLO series algorithms alongside those of the TomaFDNet model for detecting Early Blight, Late Blight, and Leaf Mold. In the **Figure 13**, white circles indicate missed detections, while red circles denote incorrect detections. The numbers within the detection boxes represent the confidence levels, which range from 0 to 1; a higher value signifies greater certainty that the model has accurately identified the target object.

It is evident from the **Figure 13** that the TomaFDNet model consistently demonstrates high confidence levels in detecting tomato diseases, outperforming other YOLO series algorithms. Specifically, for Early Blight, TomaFDNet’s detection confidence exceeds that of the original YOLOv8 algorithm by 0.14, indicating enhanced reliability. In the case of Late Blight, TomaFDNet successfully detected the disease, while the original YOLOv8 failed to identify a lesion in the middle right portion of the image (marked by a white circle), showcasing TomaFDNet’s superior sensitivity and accuracy in detecting fine lesions. Furthermore, during the Leaf Mold detection tasks, models such as YOLOv5, YOLOv9, and YOLOv10 exhibited varying degrees of misdetections and missed detections (indicated by red and white circles). In contrast, TomaFDNet not only avoided these errors but also demonstrated a stronger performance in both detection regions and confidence levels.

To further confirm the efficacy of the TomaFDNet model in identifying tomato diseases, we selected leaf images representing three distinct disease types and conducted comparative experiments. Additionally, we utilized Gradient-weighted Class Activation Mapping (Grad-CAM) technology to visualize the decision-making process of YOLOv8 and the TomaFDNet model. Grad-CAM generates heatmaps that visually highlight the areas of the image that the model focuses on during decision-making, with varying color intensities indicating the level of attention. Darker shades represent areas of greater focus by the model.

**Figure 14:**
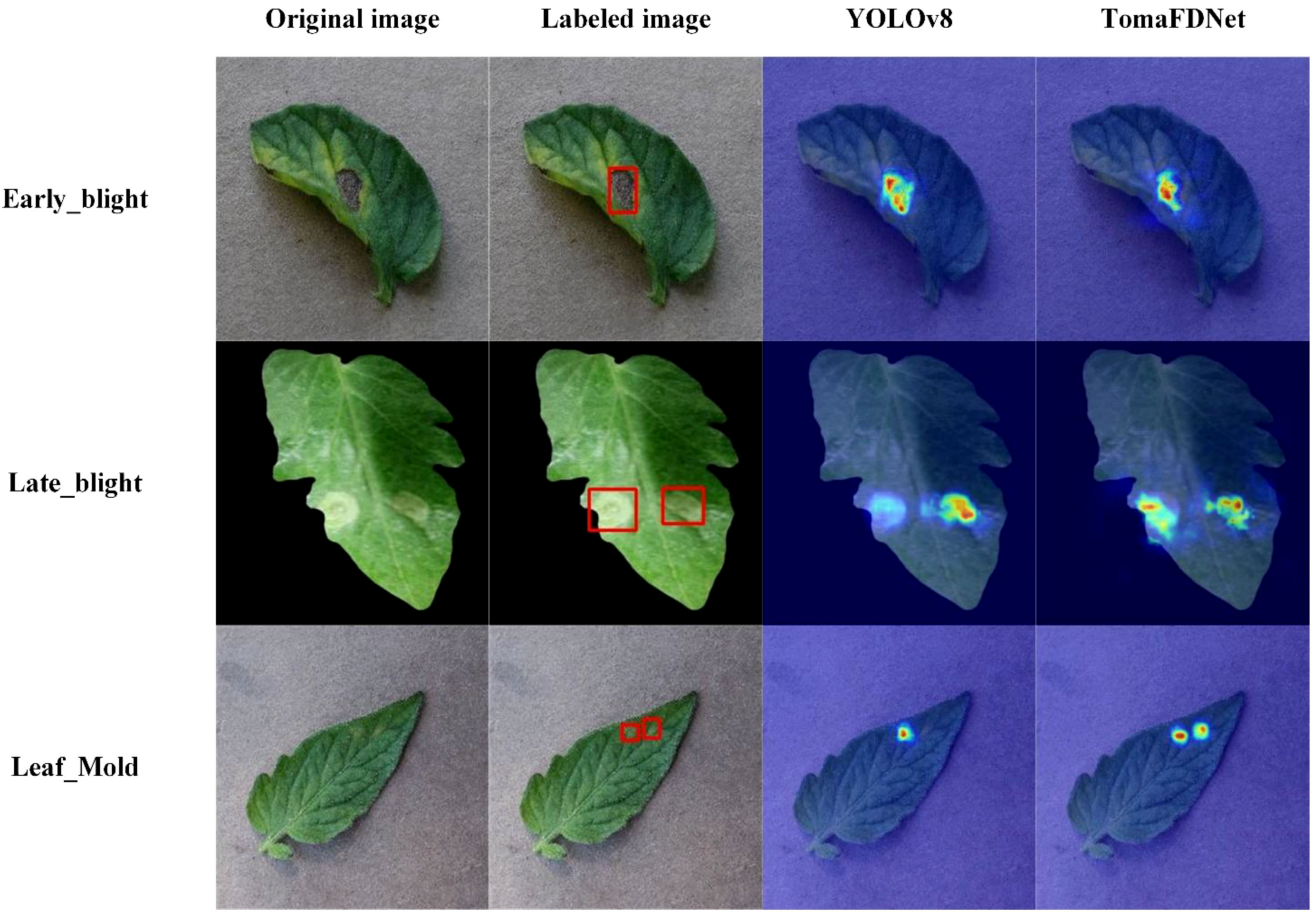
Grad-CAM comparison of different types of tomato diseases.

As shown in the experimental results in **Figure 14**, TomaFDNet exhibited superior accuracy and sensitivity in detecting disease spots compared to the original YOLOv8 algorithm. Notably, TomaFDNet demonstrated enhanced precision in accurately locating lesions of varying sizes and shapes, effectively avoiding misclassification of non-lesion areas. Furthermore, the heatmaps produced by TomaFDNet displayed stronger color responses, indicating a heightened attention to diseased regions. In contrast, while YOLOv8 was able to recognize lesions, its color responses were relatively weak, and the delineation of lesion shapes and boundaries appeared ambiguous.

We performed a comparative assessment of the TomaFDNet model against seven mainstream target detection algorithms, including Faster R-CNN and YOLO series, such as v5, v7, v8, v9, v10, and 11. The results of the experiment are displayed in **Table 6**. As evidenced by the data, TomaFDNet achieved the highest detection performance in identifying tomato leaf diseases, with a mAP of 83.1%, significantly surpassing all other models. Notably, substantial improvements were observed in the detection of Late blight and Leaf Mold. In contrast, Faster R-CNN recorded a mAP of only 68.2%. While the mAP values of the other YOLO series algorithms exceeded that of Faster R-CNN, none matched the performance of TomaFDNet. These comparative experiments demonstrate that TomaFDNet not only outperforms other detection models in terms of overall accuracy but also exhibits excellent stability and precision in identifying specific disease categories.

**Table 6:** Comparison of 7 mainstream detection models.

Methods	Params (M)	Model size (MB)	mAP	AP			
				Early_blight	healthy	Late_blight	Leaf_Mold
Faster R-CNN	137.1	108	0.682	0.504	0.986	0706	0.532
YOLOv5	7.02	14.5	0.755	0.640	0.994	0.826	0.561
YOLOv7	36.50	71.3	0.783	0.614	0.993	0.831	0.694
YOLOv8	3.01	6.3	0.789	0.686	0.994	0.826	0.649
YOLOv9	2.62	6.1	0.790	0.677	0.991	0.843	0.649
YOLOv10	2.70	5.8	0.775	0.666	0.981	0.828	0.627
YOLO11	2.58	2.5	0.792	0.692	0.994	0.825	0.658
TomaFDNet	2.66	5.6	0.831	0.708	0.993	0.870	0.753

In order to comprehensively evaluate the performance of TomaFDNet, we compare and analyze the existing research methods. As shown in **Table 7**, our proposed TomaFDNet implements 83.1% mAP while significantly reducing model complexity.

**Table 7:** Comparison of existing relevant research methods.

Method	Dataset	Disease categories	Params (M)	Model size (MB)	Performance (%)
CNN (Ferdouse Ahmed Foysal et al., 2020)	Plant village	6	/	/	76.0 (Average Accuracy)
VGG19 (Rubanga et al., 2020)	Tomato leaves	3	/	/	78.3 (Average Accuracy)
Proposed_Mask RCNN (Kaur et al., 2022)	TLDD	6	/	41.18	88.2 (mAP)
YOLO-TGI-M (Kang et al., 2024)	Tomato leaves	4	11.6	23.6	83.0 (mAP)
TomaFDNet (Ours)	TDGA	3	2.66	5.6	83.1 (mAP)

As shown in **Table 7**, the detection accuracy of VGG19 and CNN models on similar data sets is 78.3% and 76.0%, respectively. In contrast, TomaFDNet improved its detection accuracy by 4.8% and 7.1%, respectively, compared to the two models, despite targeting fewer disease categories. In terms of performance comparison, although Mask R-CNN achieved 88.2% of the mAP value, its model volume reached 41.18MB, which was significantly higher than the 5.6MB of TomaFDNet. This large model size leads to a significant shortfall in computational efficiency for Mask R-CNN, which limits its application potential in real-time agricultural scenarios. It is worth noting that although the YOLO-TGI-M model achieves a mAP value similar to that of TomaFDNet, reaching 83.0%, its model volume of 23.6MB is more than 4 times that of TomaFDNet. In addition, the number of parameters of TomaFDNet is 2.66M, and the number of parameters of YOLO-TGI-M is 11.6M, which is 77% less than that of YOLO-TGI-M, fully reflecting its advantages in lightweight design. The experimental results show that TomaFDNet not only performs well in terms of detection accuracy, but also effectively solves key problems in resource-constrained environments, which is crucial for practical agricultural applications.

### 3.3 Comparison of detection performance in different environments

**Figure 15:**
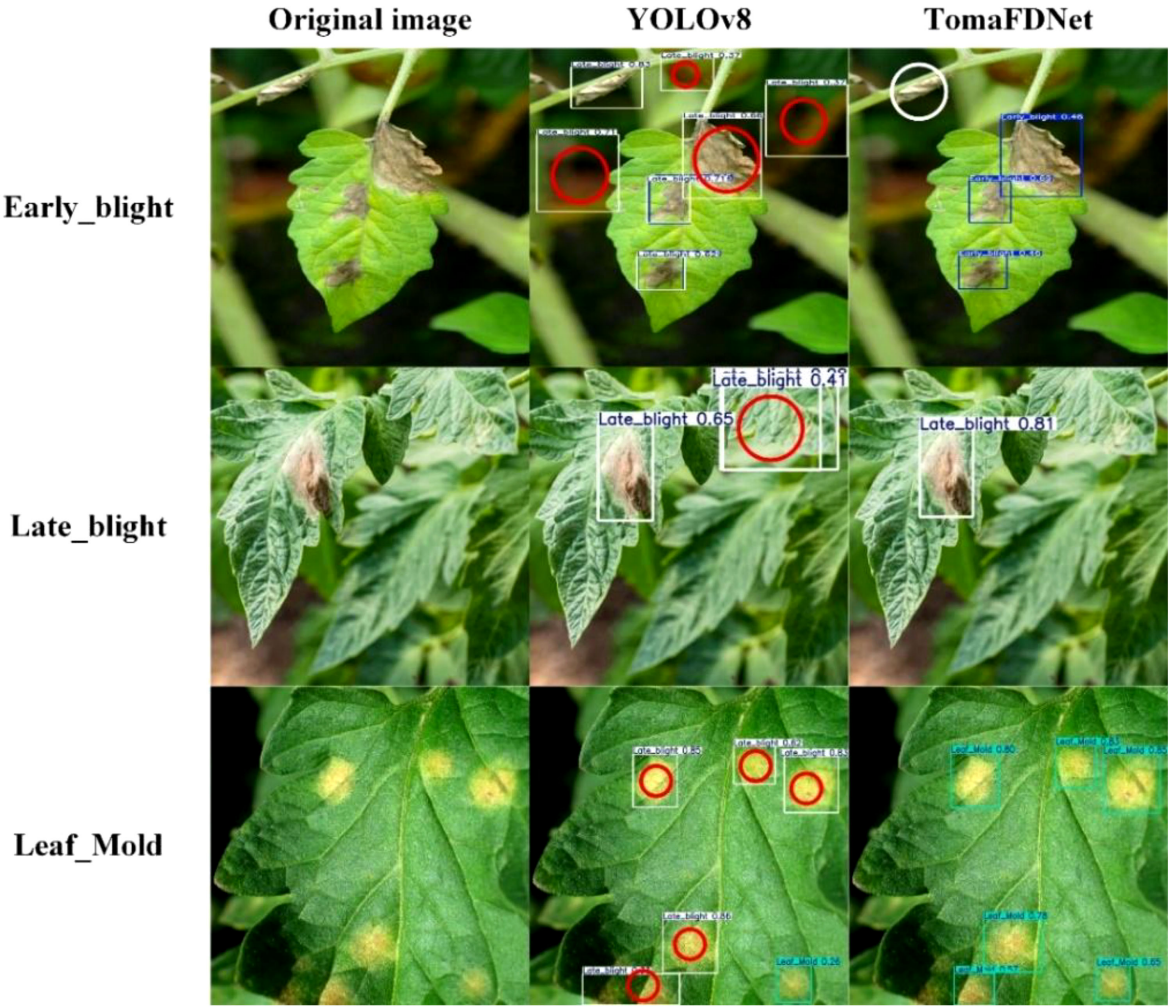
The result of the actual environment test.

**Figure 16:**
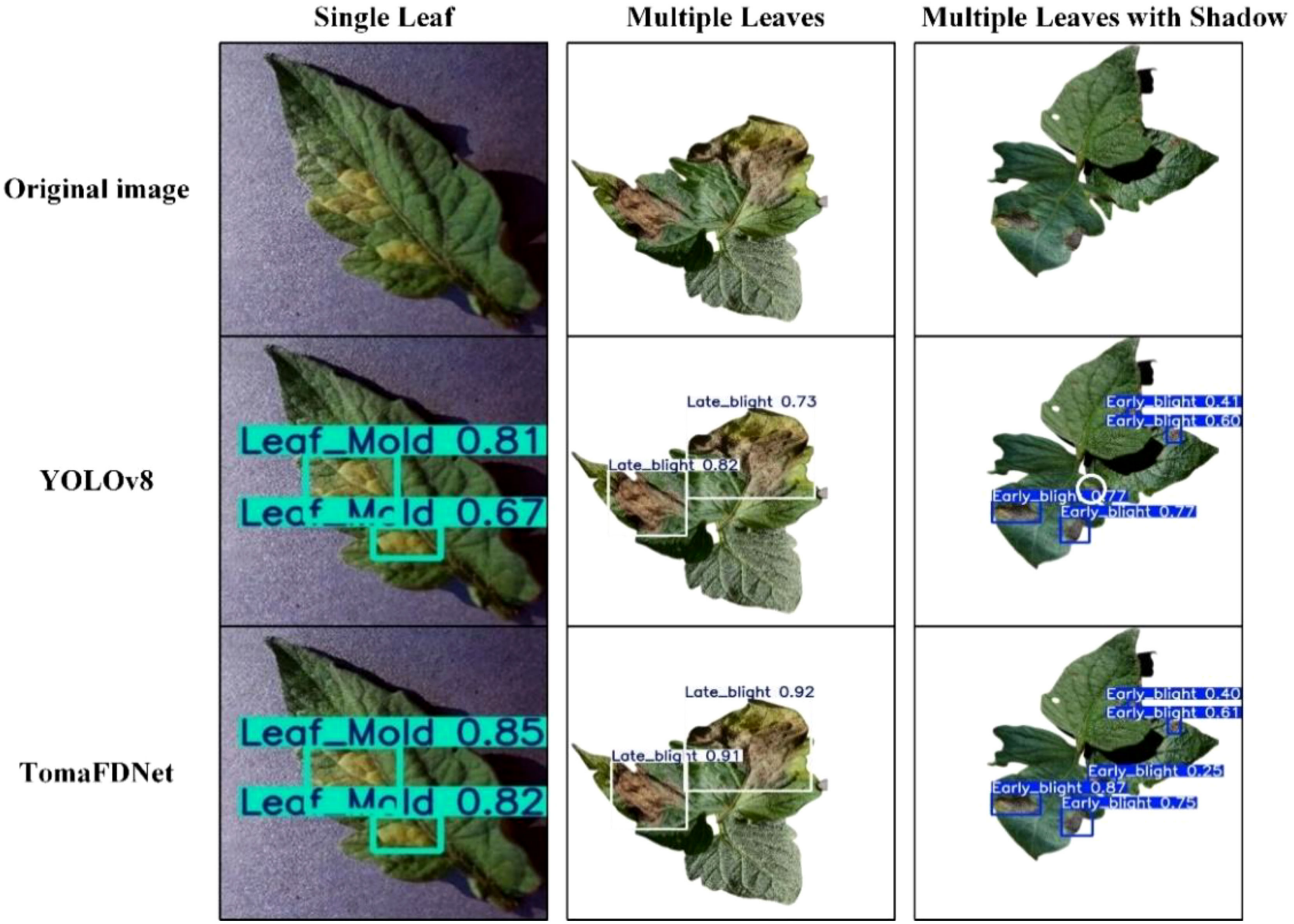
Results of different number of tomato leaves and tomato leaves with shadows.

We also assessed the robustness and generalization capabilities of the TomaFDNet model by utilizing a tomato leaf dataset sourced from the Kaggle platform (Tomato Disease Multiple Sources) under varying growth environments and shooting conditions. A selection of images depicting different scenarios, including single, multiple, and shaded leaves, was randomly chosen for prediction testing to simulate diverse lighting conditions. As illustrated in **Figures 15** and **16**, TomaFDNet demonstrated superior accuracy in detecting three common tomato leaf diseases. While TomaFDNet recorded one missed detection in the Early Blight category, the original YOLOv8 model exhibited ten false detections with significantly lower confidence scores. Notably, TomaFDNet effectively minimizes both false and missed detections, even in complex backgrounds or fluctuating lighting conditions. In scenarios involving multiple leaves and shadows, TomaFDNet’s detection results were more precise, and its confidence scores were markedly higher than those of YOLOv8. These findings indicate that TomaFDNet maintains excellent detection performance across varied environments and scenes, making it well-suited for multi-scene tomato disease detection tasks.

## 4 Discussion

In this study, we addressed the bottleneck challenges in detecting tomato leaf diseases under complex environments and recognizing small targets by designing the MSFDNet structure and EPMSC module, leading to the development of our TomaFDNet model. The results of the experiments reveal that TomaFDNet significantly outperforms the original YOLOv8 algorithm in terms of detection accuracy and robustness. The MSFDNet structure improves the model’s capabilities in two ways. First, it enhances the acquisition of multi-scale features through focused processing. Second, it uses feature diffusion mechanisms to better integrate these features. These improvements lead to better detection accuracy, particularly for small targets in complex backgrounds. Additionally, the EPMSC module further enhances the recognition capability for objects of varying scales while reducing computational resource consumption through its efficient group convolution and multi-scale convolution kernel design.

The results from the ablation experiments effectively validate the independent contributions of the MSFDNet and EPMSC modules. When only the MSFDNet structure was introduced, the model demonstrated an improvement in mean Average Precision (mAP) across various disease detection tasks, highlighting its capability to capture fine-grained features in the detection of small target diseases. The combination of the MSFDNet with the EPMSC module resulted in even more significant enhancements, achieving an mAP of 83.1%, which represents a considerable advancement compared to the original YOLOv8 algorithm. Furthermore, we conducted comprehensive comparisons with popular detection models, including Faster R-CNN, YOLOv10, and YOLO11. The results indicated that TomaFDNet consistently outperformed these models in multiple disease detection tasks. While YOLOv8 excels in detection speed, it exhibits limitations in managing complex backgrounds. In contrast, the TomaFDNet model enhances the transmission of contextual semantic information through a series of upsampling, downsampling, and feature diffusion processes. This approach not only enhances the model’s capacity to identify intricate details in small target detection but also mitigates issues of false detection that may arise from complex backgrounds or high target density.

In addition to the advancements in deep learning-based detection, recent developments in wireless sensing systems, such as Vis/NIR optical sensing and capacitive sensing, have shown great potential in agricultural monitoring (Han et al., 2024). For instance, Wang et al. (2023) demonstrated the effectiveness of Vis/NIR sensing in non-destructively monitoring fruit ripening by analyzing spectral changes, achieving high prediction accuracy. Similarly, Kim et al. (2023) proposed a low-power, wireless sensing system for real-time monitoring of tomato ripening stages, which could complement our TomaFDNet model by providing additional environmental data for disease prediction. Integrating such wireless sensing technologies with TomaFDNet could further enhance the model’s ability to detect diseases under varying environmental conditions, particularly in complex agricultural settings.

The practical deployment potential of TomaFDNet in agricultural scenarios is significant. The model could be integrated into various automated monitoring platforms. For aerial surveillance, TomaFDNet could be deployed on agricultural drones, enabling rapid disease detection across large-scale tomato fields. In greenhouse environments, the model could be incorporated into fixed camera monitoring systems for continuous plant health assessment and early disease warning. For small-scale farmers, TomaFDNet could be implemented in mobile applications, allowing real-time disease diagnosis using smartphone cameras.

Despite the remarkable results achieved by the TomaFDNet model in tomato disease detection, there are still some limitations and challenges that require further improvement and research. In the detection of some specific diseases, especially in the early stages of the disease, the features of the diseased areas may be very weak and have a low contrast with the healthy parts, which makes the model may misjudge or omit to recognize these diseases. In addition, environmental factors may also have an impact on model performance. Complex backgrounds and different lighting conditions may interfere with the identification of diseased areas. Although we have used multi-scale feature fusion and feature diffusion mechanisms in the model to enhance the detection ability under background complexity, the model still faces some challenges under extreme weather conditions (e.g., glare, shadow, haze, etc.) or harsh environments (e.g., moisture, dust). Especially in agricultural environments, plant shading and overlapping may make it difficult to correctly identify certain small disease areas.

Future research will focus on improving the performance of the TomaFDNet model for specific disease detection and environmental adaptation. We plan to introduce higher resolution image acquisition techniques and incorporate more deep learning methods to further improve the recognition of early disease features. To cope with the limitation of computational resources, future research will also focus on the lightweight design of the model and optimize the model structure so that it can run efficiently on resource-limited devices to ensure its usability and real-time performance in practical agricultural applications. In addition, we will explore how to integrate wireless sensing technologies, such as visible/near-infrared sensing and capacitive sensing, to provide real-time environmental data to further enhance the robustness and accuracy of agricultural disease detection systems.

## 5 Conclusion

In this study, we proposed the TomaFDNet model, a multi-scale detection method for tomato diseases based on an improved YOLOv8 architecture. By integrating the MSFDNet and EPMSC modules, the model significantly enhances its ability to capture multi-scale features and improve robustness in complex environments. The experimental results demonstrate that the mAP of the TomaFDNet model is superior to that of several mainstream models, achieving an impressive mAP of 83.1%. This represents increases of 14.9%, 7.6%, 4.8%, 4.2%, 4.1%, 5.6%, and 3.9% over Faster R-CNN and various YOLO series algorithms (v5, v7, v8, v9, v10, and 11), respectively. Furthermore, predictive experiments conducted in diverse environments revealed that the TomaFDNet model can accurately detect tomato diseases across different scenarios, underscoring its effectiveness in disease detection. Future work will focus on developing a lightweight version of the model to enhance detection speed and real-time performance.

## Data Availability

The original contributions presented in the study are included in the article/supplementary material. Further inquiries can be directed to the corresponding author/s.
